# Hybrid Cooperative Complexes of Low- and High-Molecular-Weight Hyaluronic Acid in Aesthetic Medicine

**DOI:** 10.3390/ph19010073

**Published:** 2025-12-30

**Authors:** Goran Tintor, Tin Cohadzic, Josipa Bukic, Dario Leskur, Lovre Zekan, Doris Rusic, Mladen Dudukovic

**Affiliations:** 1 Department of Surgery, University Hospital of Split, 21000 Split, Croatia; gogitintor@gmail.com; 2 Department of Paediatric Surgery, University Hospital of Split, 21000 Split, Croatia; tin.c94@gmail.com; 3 Department of Pharmacy, University of Split School of Medicine, 21000 Split, Croatia; dleskur@mefst.hr (D.L.); lovre.zekan@ljekarnasdz.hr (L.Z.); drusic@mefst.hr (D.R.); 4 Department of Laboratory Medicine and Pharmacy, Faculty of Medicine, Josip Juraj Strossmayer University of Osijek, Josipa Huttlera 4, 31000 Osijek, Croatia; 5 Laboratory for Quality Control of Galenic Preparations and Identification of Medicinal Substances, Split-Dalmatia County Pharmacy, 21204 Dugopolje, Croatia; 6 Dudukovic Kisic Malic Polyclinic, 10000 Zagreb, Croatia; mladen.dudukovic@hotmail.com

**Keywords:** hybrid cooperative complexes of low- and high-molecular-weight hyaluronic acid, hyaluronic acid, hyaluronan, skin booster, dermal filler, aesthetic medicine

## Abstract

In this review we present a comprehensive overview of the published literature related to the use of Hybrid Cooperative Complexes (HCCs) of low- and high-molecular-weight hyaluronic acid in aesthetic medicine. HCCs have been developed to overcome the shortcomings of traditional hyaluronic based dermal fillers. Specifically, HCCs deliver both high- and low-molecular-weight hyaluronic acid (HA), maximizing their complementary effects. They are biocompatible and formulated without the addition of foreign agents. Cooperative hydrogen bonds extend their durability and make them more resistant to hyaluronidase compared to high-molecular-weight HA. The rheological properties of HCC formulations allow for easy exertion through the needle and diffusion in the tissue compared to high-molecular-weight HA alone. In vitro studies have shown that HCCs improve vitality of fibroblasts, keratinocytes and adipocytes, and stimulate production of collagen and elastin. Studies on scratched co-cultures of immortalized human keratinocytes and human dermal fibroblasts demonstrated that HCCs accelerate wound closure. Furthermore, HCCs delayed senescence of mesenchymal stromal cells to a greater extent than high-molecular-weight HA or low-molecular-weight HA alone. Clinical studies show a reduction in wrinkle severity, improvement in skin roughness profile and reduction of skin laxity with pronounced improvement in superficial skin hydration lasting up to 6 months. The formulation intended for restoration of fat compartments demonstrated reduction in cheek volume loss and improvement in skin thickness. Subjects report moderate-to-high satisfaction and are likely to recommend the treatment. Limitations of the published studies are also addressed, as well as reported adverse events and published safety data.

## 1. Introduction

Modern society is visual and takes many cues from one’s appearance into account; it comes as no surprise that facial rejuvenation is among the most sought-after treatments [1,2,3]. Aging is a complex process, ceaseless in nature, that affects the entire body, including skin, affecting its appearance and function [4]. Skin aging affects not only the face, but also various areas of the body, which includes the neck, décolleté, arms, knees and abdomen. As individuals age, their skin tends to become rougher and exhibit increased laxity due to more pronounced sagging [4,5]. Consequently, maintaining a youthful appearance is highly valued, with the preservation of such features becoming a central focus within aesthetic medicine. Procedures that help restore youthful look are in great demand and rising every year [6,7,8,9].

One of the treatment options to address these concerns is implantable medical devices—injectable dermal fillers that include a number of products with different chemical composition, mechanisms of action, interactions with the host tissue, durability and safety profiles [8]. Fillers also differ based on the method of application in terms of the device used (needle or cannula) and the injection depth, for different desired outcomes [8]. To restore volume, fillers are mostly injected into deep skin layers, either in the deep dermis or in subcutaneous fat [8].

However, even the field of aesthetic medicine recognizes that immediate correction alongside long-term dermal remodeling is necessary for rejuvenation, hence why it is shifting towards treatments that enhance skin’s intrinsic repair mechanisms [2,10,11,12,13]. Skin boosters, a type of dermal filler, are injected into the dermis to hydrate and revitalize the skin for a smoother, healthier appearance [8].

## 2. Hyaluronic Acid in Aesthetic Medicine

### 2.1. Biological Role and Molecular Weight-Dependent Effects

Several products are classified as dermal fillers, but one stands out because it is used not only as a filler but also as a skin booster in various formulations—a naturally occurring glycosaminoglycan composed of alternating units of D-glucuronic acid and N-acetyl-D-glucosamine disaccharide bound with beta-1,4 and beta-1,3 glycosidic covalent bonds: hyaluronic acid (HA) [7,8,14,15,16,17,18,19]. This linear anionic polysaccharide is sometimes referred to as hyaluronan to identify the polysaccharide irrelevant of its degree of dissociation [7,14,17,20].

HA is the key factor in the most popular non-invasive restoration approaches in aesthetic medicine leading to rejuvenation effects, and is high in demand for age-related soft tissue defects, recognized as critical for skin aging [7,21,22,23,24]. Senescent skin is characterized by diminishing epidermal HA alongside progressive shrinking of the HA polymers [7,25], and photo-exposed skin has a decrease in the expression of hyaluronan synthases and increased expression of hyaluronidases [7,26]. Skin aging results in decreased fibroblast activity, reduced synthesis of dermal extracellular matrix components, decreased production of HA and collagen, leading to decreased elasticity and turgidity [4,16,27]. In short, aging alters mainly HA content, but also elastin and collagen content, in the skin [23,28].

On average, a person has 15 g of HA, of which 50% is found in the skin, one-third of which is degraded and synthesized daily [14,29,30,31]. In the skin, HA is found in the basal layer of the epidermis with proliferating keratinocytes [14,32]. In the epidermis, HA maintains extracellular structure supporting the components of the extracellular matrix—mostly fibroblasts—and controls tissue hydration and permeability [14,23,33].

HA may restore normal dermis function and is used to counteract skin aging [23]. Part of the reason is that HA has a leading role in maintaining skin moisture [7,25]. Acidic functional groups of HA hydrogels are ionized at physiological pH. This causes the polymer to expand as charged carboxylate anions repel one another. Molecules of water are then attracted and trapped in the charged groups [14,32]. In this way it enhances volume and provides structural support [4,34]. HA improves skin hydration and skin elasticity, and boosts collagen production, counteracting the negative effects of aging on the skin [4,34,35]. Moreover, during their lifetime, fibroblasts can be stimulated to produce new collagen by HA via mechanical stretching of the dermis, as shown with cross-linked HA [14,36,37]. HA has stimulating effects on dermal cellular and extracellular components, including elastin production and expression of growth factors [37,38,39].

Extracellular polymers of HA that are 104 kDa in size have hydrating and space filling properties, adjunct to immunosuppressive and anti-angiogenic effect. Small oligomers have anti-apoptopic functions and polymers under 5 kDa have immunostimulatory, inflammatory and angiogenic effect [7,9,40,41,42,43].

Furthermore, it exhibits wound healing capacity [33,44,45]. HA takes one of the lead roles in tissue injury, as its synthesis spikes leading epithelial cells’ and fibroblasts’ response to injury [7,16]. By propagating inflammatory response, it induces macrophages and chemokine response. These effects contribute to the healing process [27,46,47,48]. In the initial stage of wound healing, there is a surge of high-molecular-weight HA (H-HA). H-HA leads to aggregation, accumulating and binding of fibrinogen for clot formation. This great amount of H-HA opens up tissue spaces, facilitating the activity of polymorphonuclear leucocytes at the wound site to remove dead tissue, debris and bacteria. Furthermore, it demonstrates a great water retention capacity and interacts with proteoglycans and collagens to alter the dermal structure [49,50,51]. In the inflammatory stage of wound healing, low-molecular-weight HA (L-HA) builds up after the degradation of H-HA and induces cytokine response with angiogenesis [33,49,52].

HA activates different signaling pathways through interaction with CD44, toll-like receptor 4 (TLR4) or receptors for hyaluronan-mediated motility (RHAMM). Furthermore, research demonstrated HA’s ability to prevent reactive oxygen species (ROS)-induced damage. Based on molecular weight, HA may exhibit distinct physiological effects [23,45,53]. H-HA stimulates CD44 signaling and clustering that masks their death, preventing cell apoptosis; it inhibits endothelial cell proliferation, motility and sprout formation in anti-angiogenic response and reduces oxidative stress. L-HA has been shown to disrupt CD44 clustering and inhibit kinase activation, stimulate production of pro-inflammatory cytokines, chemokines and growth factors, stimulate vascular endothelial cell proliferation, migration and tubule formation and inhibit free radicals. Both H-Ha and L-HA have demonstrated stimulation of fibroblast proliferation and migration via RHAMM. In interaction with TLRs, HA regulates cell response to pathogens. The H-HA protects the cell surface TLRs from the outside. H-HA via TLR suppresses inflammatory cascade in acute injury, diminishes recruitment of inflammatory cells and decelerates migration of stem cells, thus preventing the cellular inflammatory response. The L-HA, on the other hand, provokes inflammatory response, activating TLRs to produce pro-inflammatory cytokines as well as chemokines [9,43].

### 2.2. Conventional HA Fillers and Skin Boosters: Limitations

HA has been in the category of dermal fillers for decades and has been undergoing different modifications ever since to improve its stability, enhance desirable effects and reduce the likelihood of adverse events [8]. HA is considered to have minimal immunogenicity since it does not have protein epitopes (unlike collagen) and, due to the fact that it is an integral part of the extracellular matrix, is a component of human connective tissue. As such, HA-based injectables do not require allergy testing [6,7,8,23,38,50,54,55,56,57,58,59,60].

There are different preparations of HA available based on different characteristics such as concentration and consistency profiles, and HA hydrogels can be formulated with different molecular weights, at different concentrations [61,62,63,64]. The major shortcoming of the linear HA is its short durability in vivo [65]. As HA is natural in the human body, it has its natural antagonist family of enzymes, hence making it biodegradable [8,50,61]. Hyaluronidases catalyze the degradation of HA, reversing its effects and turning the native H-HA into L-HA fragments [7,8,42]. This makes HA especially appealing since the results may be easily reversed in the case of adverse events [7,54]. Therefore, after being injected into the dermis, HA is no longer viable [66,67]. To overcome this, scientists developed various formulations of chemically stabilized HA, cross-linked HA increasing the rigidity, elasticity and stability of HA [66,68]. This modification of the natural structure leads to improvement in stability, rigidity and elasticity as the fluid is transformed to gel that is less water-soluble, hence stable in living tissues [23,69,70,71].

Different formulations of HA fillers with different degrees of cross-linking are available to ensure longer results and higher density of the fillers [8,62,63,72]. Cross-linked HA exhibits greater toxicity and is not a guarantee of sustained improvements over time, as it does not alter the biological and cellular function as does the endogenous HA [51,73,74]. As previously noted, HA exhibits a relatively brief half-life following injection. Consequently, multiple strategies have been implemented to enhance its stability and duration of action, as well as to improve degradation control and release properties. One of the ways to achieve this was by chemical modifications of the structure of HA with various cross-linking agents. Divinyl sulphone (DVS) and butanediol diglycidyl ether (BDDE) were the most commonly used agents [5,75]. Use of Hybrid Cooperative Complexes (HCCs) was an alternative way to achieve higher resistance to degradation [63]. Another approach included development of novel composite hydrogels. In a study by Fan et al., HA—hydroxyapatite hydrogels—showed better longevity than HA alone, with improved collagen synthesis and elastic fiber regeneration [76]. Similarly, complex hydrogels based on physical interactions between gelatin, HA and cationic cellulose nanocrystals were developed and proposed as a new material for dermal fillers [77]. The incorporation of nanoparticles into HA–based formulations can enhance product longevity. An aforementioned study by Fan et al. demonstrated that HA hydrogels containing nanospheres of hydroxyapatite had better stability than the pure HA and HA–microsphere hydroxyapatite hydrogels [76]. An additional option could be the use of stimulus-responsive hydrogels as dermal fillers. Those are hydrogels that change their state depending on external factors such as temperature, pH, etc. [78,79]. This would allow the injection of filler in sol state, allowing easier administration. After injection, it would solidify to gel [80]. Such HA-based gels were already developed, albeit for different applications, such as drug delivery systems for controlled drug release, but they could be adapted for future use as dermal fillers [80,81,82].

## 3. Hybrid Cooperative Complexes (HCCs) of Low- and High-Molecular-Weight Hyaluronic Acid

### 3.1. Rationale and Technology

Since cross-linked HA does not fully mimic the endogenous HA, and as H-HA and L- HA have complementary effects on skin function, it was prudent to combine the H-HA and L-HA to maximize their synergistic effect on tissue regeneration. Research indicates that Hybrid Cooperative Complexes (HCCs) of low- and high-molecular-weight hyaluronic acid stimulate the proliferation of fibroblasts and keratinocytes and have both anti-inflammatory and biostimulating effects [23,63]. Following this approach in 2015, there was a launch of a novel formulation of L-HA (80–100 kDa) and H-HA (1100–1400 kDa) stabilized Hybrid Cooperative Complexes (HCCs) [83]. It was formulated in a unique manner with new patented NAHYCO™ hybrid technology to enable the product to spread in the skin, allowing for fewer injections, usually in two sessions 4 weeks apart [33,84]. The results are expected to last up to 6 months [4,6,8,27,59,85,86,87]. The patented technology of two-step thermal stabilization process that involves a phase of controlled heating succeeded by a phase of controlled cooling results in hydrogen bonds between the molecules with no addition of cross-linking chemicals [23,86].

To produce HCCs, one needs HAs of specific molecular weights (MWs) in equal concentrations in a solution. The solution is first heated to 100–120 °C for roughly 10 min. This causes the disruption of the hydrogen bonds. After the rapid cooling to room temperature, the bonds start to rebuild in random manner, creating stabilized cooperative complexes. Typically, weak hydrogen bonds are highly stable when in cooperative complexes relative to the length and concentration of HA chains in the solution. This rearrangement influences the rheological properties of the material, as reflected in a reduction in viscosity [63,88,89,90].

### 3.2. Rheological Properties

A marked reduction in zero-shear viscosity (η_0_), which represents the viscosity of the material at very low shear rates and reflects polymer chain interactions at rest, is observed after thermal processing. This decrease indicates reduced molecular entanglement and supports the formation of the hybrid network structure. When H-HA alone (16 mg/L), a mixture of L-HA and H-HA (16 + 16 mg/L) and HCCs (16 + 16 mg/L) were exposed to thermal treatment of 120 °C during 12 min, there was a marked 21.9-fold decrease in dynamic viscosity for HCCs, indicating complex formation with a final value of 5.5 ± 0.0 Pa s. This drop is related to the capacity of intermolecular interactions [23,86]. For H-HA alone (16 mg/mL), the change of zero-shear viscosity η0 pre- to post-treatment resulted in a 3.2 decrease, with a final value of 23.0 ± 0.2 Pa s, and, for the mixture of the two MW HAs, there was a 4.7-fold decrease to the value of 28.3 ± 0.4 Pa s. This low value of η0, reduced dynamic viscosity, gave the product enhanced injectability, delivering very high concentration, and allowed for better diffusion of the product within the tissue [6,23]. It was suggested that the complexes release smaller chains over time, thus triggering biochemical repair [33].

Another study investigating HCCs of 1600 kDa H-HA and 220 kDa L-HA prepared with the patented technology reported that post-thermal-treatment H-HA has zero-shear viscosity η0 values of 94.73 ± 0.46 Pa s, L-HA 3.95 ± 0.01 Pa s and HCCs 16.60 ± 0.06 Pa s [91]. The unique biophysical profile of predominant fluidity over elasticity enables the product to uniformly diffuse in various tissues or layers and to homogenously expand adipose compartments [6].

Rheological assessment is essential for injectable HA fillers and skin boosters because viscoelastic properties influence their injectability, tissue integration, spreadability and clinical performance. During oscillatory testing, two key parameters are measured: the storage modulus (G′), which reflects the elastic, solid-like response; and the loss modulus (G″), which reflects the viscous, liquid-like response of the material. Tan δ (G″/G′) describes the balance between viscous and elastic behavior [92].

Since HCCs contain a higher HA concentration compared to other skin boosters, they exhibit a higher storage modulus (G′) and loss modulus (G″), reflecting a more structured and cohesive viscoelastic network while retaining flowability. The tan delta (tan δ), also called the loss angle or dissipation factor, describes the ratio of viscous to elastic response in a viscoelastic material and describes how it behaves under mechanical stress. A tan δ value approaching 0 indicates a predominantly elastic, shape-maintaining behavior, while very high values approaching infinity indicate a predominantly viscous, fluid-like behavior. HCCs have tan δ values slightly above 1, indicating sufficient fluidity for easy distribution in superficial tissues. On the other hand, their relatively lower tan δ compared with other non-cross-linked boosters reflects a modest but important elastic component, which contributes to cohesivity, tissue support and resistance to mechanical stress. This balance prevents excessive spread while allowing natural integration, making HCCs suitable for biostimulatory and hydration-focused treatments rather than projection or lifting [92].

This allows for improved potency of the product and enables a higher concentration of HA (L-HA and H-HA, 32 mg each in 2 mL, 3.2% concentration) to be delivered compared to previously developed cross-linked HA fillers [27,93]. The bonds make the HAs more resistant to the effect of hyaluronidase and extend the persistence of the product in the skin [23,83]. After one day of incubation of HCCs with Bovine Testicular Hyaluronidase (BTH), the fraction of the H-HA was reduced to about 35% of the initial amount, while, for the H-HA alone, it was less than 10% of the starting concentration. No significant degradation was observed after this period during the 10-day incubation [23]. A similar study found that in the first 2 h of incubation, H-HA reduction was 44% for H-HA alone and 11% for the complexes prepared with 1400 kDa MW H-HA and 90 ± 5 kDa L-MW HA. After 24 h, the high-molecular-weight fraction in H-HA reduced by 50% and only 6.25% in HCC [33]. Moreover, the HCC formulation is stable and completely free of the classic cross-linking agents such as 1,4-butanediol diglycidyl ether [66,94].

The product has a number of advantages other than extended half-life, including lower inflammatory response due to the fact that Transforming Growth Factor Beta 1 (TGF-β1) is less upregulated (4 times lower than with L-HA) and assumed greater safety due to absence of cross-linking chemicals [33,83,84]. It is appealing as the clinical studies reported early visible effects with minimal downtime, due to the fact that it provides immediate results with short recovery time [66].

Different products were marketed with 3.2% HCCs, one to be applied in the face and the other in the body to address the aesthetic concerns in other areas and formulated for superficial intradermal injection. The latter comprises both low- and high-molecular-weight HA, 48 mg each in 3 mL (3.2% HCC the body formulation) [95,96]. As changes in facial subcutaneous adipose tissue structure and volume contribute to aging appearance [95,97,98], a more concentrated formulation has been developed for the restoration of adipose tissue with the same technology and containing 45 mg/mL (4.5%) of both L-HA and H-HA in 2 mL. This higher concentration has direct impact on the product’s rheological properties, cohesivity and resistance to BTH, which is now comparable to those of cross-linked HAs [51,99,100,101]. The new formulation has a great ability to preserve its structure (higher G’ compared to 3.2% HCCs) in the tissue with no alteration to spreadability (slightly lower tan δ compared to 3.2% HCCs) [100]. An in vitro enzymatic degradation study confirmed similar degradation kinetics for 4.5% HCCs and cross-linked HA, but it was completely depolymerized after 3 h compared to the cross-linked product, for which there was a delay, and it took 24 h for the same degree of depolymerization, indicating that HCCs can be quickly degraded if needed, adding to the safety of the product [101]. It is formulated for integration in superficial fat compartments [95,100]. In another study, after 5 min of incubation with BTH 77% (G’) and 87% (G’’) of 4.5% HCCs, elastic and viscous moduli were preserved, while H-HA maintained only 50% and 68% of initial values. In the prolonged hydrolysis after 25 min, 38% G’ and 55% G’’ were preserved for the HCCs compared to 5% G’ and 15% for the H-HA, demonstrating that 4.5% HCCs are able to better retain viscoelastic properties when exposed to hyaluronidases and are 5 to 7 times more stable than 4.5% H-HA [102]. G′ is representative of the product stiffness, referring to the ability to withstand elastic deformation during injections and capacity to maintain the physical structure when subjected to tissue movements following application. The parameter of dynamic sheer, viscosity η, is related to the hydrogel’s capacity to withstand shear forces induced by the needle during the implantation process and the physiological forces in vivo following the implantation [102,103,104]. At this high concentration, the product behaves as viscous liquid with superior mechanical performance in terms of reduced deformation under external forces compared to HCCs of lower concentration [102].

HCCs exhibit distinct rheological properties that overcome the usual downfalls of HA and cross-linked fillers [6,63,101]. Another option for longer duration without chemical modification would be to inject H-HA in great concentration, but this is not ideal due to high viscosity that requires high extrusion force from the syringe. For injectables, viscosity is a known critical parameter for mechanical and biological properties [23]. The technology of stabilized hybrid HA complexes allows for a delivery of a greater concentration of HA. HCCs, as it was described previously in this text, demonstrate low viscosity, high flowability and easy extrusion from a syringe, because of predominance of fluidity over elasticity. These properties, alongside their excellent tissue diffusion and the fact that, once injected, it integrates more naturally than synthetic injectables, result in a natural-looking effect [6,7,33,63,66,100,105]. Confirmation of these properties was obtained through a 3D camera system fifteen minutes following application of the product to the neck. The 3.5% HCCs easily distributed within dermal tissues and maintained its cohesive properties [21].

In the early years after the launch of HCCs, the number of users has doubled each year [85]. According to sales data, there is a continuous trend of increasing sales of HCCs observed from 2018 to 2023 [4,106]. Furthermore, the use of HA injectables has risen by 29% from 2022 to 2023, and according to International Society of Aesthetic Plastic Surgery, HA remains the second most popular non-surgical procedure (after botulinum toxin) for men and women of all age groups [31,107,108].

As the leading nonsurgical alternative for facial rejuvenation, soft tissue filler should be nontoxic, nonimmunogenic, nonmigratory, noncarcinogenic, easy to apply, nonpalpable, long lasting, affordable, painless and easy to remove [7,85,109,110,111]. In this review we summarize the evidence of HCCs after roughly 10 years in clinical practice across non-clinical studies, clinical studies, patient satisfaction and safety to answer the question as to whether this this truly the ideal injectable in terms of the interplay of rheology, biocompatibility, ease of use and lasting effect.

## 4. Nonclinical Evidence—In Vitro and Animal Studies

In animal models, implants generated under mouse skin with 3.2% HCCs were detectable for 10 weeks and 4.5% HCCs for 29 weeks. For reference, cross-linked HA was detectable for at least 33 weeks, while linear HA for just 4 weeks. In terms of volume, a slight decrease in volume (20%) was observed in the first 4 weeks for 3.2% HCCs, but for 4.5% HCCs, there was minimal volumetric degradation with no volume decrease in the subcutaneous implants in the first 3 weeks. A decrease of 20% was observed at week 8. After the injections, the HCC formulations led to subcutaneous ovoid bulges which subsided in the following weeks, increasing the turgor and achieving filling effect. This confirms the desired adaptability of the product to surrounding tissues and the plasticity of the formulation. On the other hand, after the injection of cross-linked HA, the bulges persisted for the entire observed period [101].

The intrinsic antioxidative capacity of the marketed product was demonstrated with FRAP, the measurement of ability to reduce Fe^3+^ to Fe^2+^; ORAC assay, measuring ability to protect a target molecule from oxidation by peroxyl radicals; and CUPRAC, measuring the ability to reduce cupric ions (Cu^2+^) to cuprous ions (Cu^+^) [92,112]. Biostimulating effects are often evaluated by measuring stimulation of total collagen [113]. After 72 h incubation with primary dermal fibroblasts, the measurements of total collagen production were significantly increased compared to control, while there was no decrease in the viability of the studied cells [92,113].

A number of in vitro studies were conducted comparing the effects of HCCs prepared with the patented technology with both H-HA and L-HA. In cultivation of mesenchymal stromal cells, onset of replicative senescence is a major obstacle. Senescent cells negatively affect their surrounding cells, releasing paracrine factors. After 30 days of incubation with 0.16% HCCs, there were 9.0 ± 1.6% senescent cells compared to 39.1 ± 2.8% senescent cells in the control group. HCC delayed senescence to a greater extent than H-HA or L-HA and promoted adipogenic and chondrogenic differentiation. No alteration to the long-term cell cycle, vitality, function, apoptosis or cytotoxicity was observed. There was a decline in cycling cells and an increment in resting cells observed, with cells being in quiescence rather than in senescence [63,114].

HCCs increase the biosynthetic capacity of fibroblasts, stimulating synthesis of new extracellular compounds [7,23]. A published study demonstrated a marked increase in the expression levels of collagen type I and II in both keratinocytes and fibroblasts and collagen type IV and VII mostly stimulated in keratinocytes with HCC. Increased elastin expression in cells was confirmed in a skin model, indicating improved skin elasticity. There was a different modulation of mRNA expression based on cell type. A significant increase in gene expression in the cell monolayer for collagen I, III, IV and VII and elastin in keratinocytes after 4 h was observed. Furthermore, increased expression of collagen I and III and elastin in fibroblasts after 24 h compared to other HA samples was confirmed. Immunofluorescence staining confirmed significant increase in elastin and collagen I and III. A full-thickness skin model of multilayer epidermal keratinocytes and dermal fibroblasts confirmed increased expression for collagen I, IV and VII and elastin after 24 h, and collagen I, III and VII and elastin after 7 days [23,63].

It is established that HCCs promote wound healing. In one study, HCCs containing H-HA (1600 kDa) and L-HA (220 kDa) were compared to L-HA, H-HA and control samples. To simulate the wound-healing process, scratched co-cultures of immortalized human keratinocytes and human dermal fibroblasts were studied. In particular, the gene expression of an antimicrobial peptide HBD-2 in the absence of its usual inflammatory stimulus. It was found that expression of HBD-2 rises 35-fold with L-HA and 100-fold with HCC (*p* ˂ 0.01). Anti-inflammatory properties of H-HA were confirmed when IL-1β was added but IL-8 expression was reduced in H-HA, and more intensively in HCC-treated cells [91]. To simulate wound closure, scratch closure was observed. After 15 h, 75 to 85% ± 3% of the scratched area was repaired in the presence of HCCs, while less than 60% ± 2% of the scratched area was repaired in other samples. HCCs encouraged wound closure after 20 ± 2 h, while it took more than 40 h in other investigated samples. L-HA was slower than control in the repair process. Faster wound closure in IL-1β-treated cells with HCCs and H-HA was further confirmed with the gene expression of gelatinase MMP-9 regulating epithelial cell migration [33,91,115]. Expression of TNF-α, IL-8 and IL-6 was reduced in the presence of any of the HAs, while there was no observed effect for interferon γ, IL-2 or IL-4. Furthermore, the researchers found that the production of elastin proteins were upregulated by both H-HA and L-HA, but to the highest degree when HCC was present indicating skin restoration. Considering the effects on wound closure and the hydrodynamic and rheological properties of HCCs, they may be suitable for specific wound treatments. They will not interfere with the natural cell migration; they are more easily spreadable compared to H-HA as the η0 of the investigated HCC was 16.60 ± 0.06 and H-HA 94.73 ± 0.43, but will retain longer at the site compared to L-HA. Moreover, preservation is retained due to greater resistance to hyaluronidases [91].

A similar study investigated HCC prepared with the patented procedure using 1400 MW kDa H-HA and 90 ± 5 kDa L-HA. Wound closure on a spontaneously transformed non-tumorigenic human keratinocyte cell line and human dermal fibroblast cell monolayers occurred after 18 ± 1 h when treated with 0.1% HCC and after 26 ± 2 h when treated with 0.1% H-HA or 0.1% L-HA. After 20 h, any treatment with HA led to 85 to 100% wound closure, while in the control sample, the closure was at 75%. The more concentrated complex, 1% *w/v* HCC, was tested on a wider scratch ˃1 mm and the closure was 5 h faster. Early on, the higher viscosity of the more concentrated HCC (η0 = 18.0 ± 1.00 vs. 1.65 ± 0.04 for the 0.1% HCC) hampered cell migration, but, as the degradation of H-HA started, the mixture likely became less viscous and the reparation occurred. The lower viscosity of the complexes allows for delivery of greater concentration of HA to the wound site. The wound closure improved 1.4-fold in the presence of HCCs when compared to the control, while in the presence of other HAs (H-HA or L-HA), it was 1.2 times higher [33].

Furthermore, it seems that the formation of complexes enhances the activity of H-HA. The hydrogen bonding allows for the small chains to release over time, making the complex a slow-release system. The L-HA would be released earlier and activate healing. This is demonstrated in the modulation of TGF-β1 gene expression. After 4 h of expression, TGF-β1 was downregulated in cells treated with HCCs or H-HA alone; expression increased after 10 and 16 h and then, within 24 h, it was comparable with control. L-HA upregulated the expression of TGF-β1 4-fold in the first 4 h. The gene expression of MMP-2 was markedly higher in the HCC samples at 10 h (16-fold higher than control), reaching maximum at 16 h (22-fold higher than control), while the maximal upregulation of MMP-9 occurred at 10 h. Upregulation of gene expression of MMP-13, involved in degradation of interstitial collagen I, II and III [33,116] was most stimulated with H-HA at 24 h (15-fold compared to other HA treated samples). Zymography revealed that active forms of MMP-2 and MMP-9 were upregulated with H-HA and HCCs. Western blotting showed a significant increase in MMP-13 protein level in HCC-treated samples. The findings of the same study further confirmed that complexes increase matrix degradation during the wound repair process. The modulation of metalloproteases alongside reduced inflammatory biomarkers suggests reduced risk of scar formation [33,63].

HCCs (16 H-HA + 16 g L-HA) boosted Nd-YAG laser in in vitro tests of wound healing on keratinocytes, with HCC reparation occurring within 24 h. Time to 80% closure was 20 ± 2 h in HCC with laser compared to 29 ± 1 h with H-HA and laser and 37 ± 1 with laser alone. In the control, it took more than 44 h for 80% closure, while in HCCs alone, it took 20 ± 1 h. There was 4 x upregulation of integrin αV, 14-fold up regulation of integrin β3 and 72% upregulation of AQP3 gene compared to 61% with laser alone [117]. For reference, wound closure is delayed in AQP3-defficient keratinocytes, and the skin of AQP3 deficient mice shows reduced elasticity, hydration and delayed barrier formation [117,118,119,120]. Integrins regulate all processes of wound healing [121]. Dysregulation of integrin β3 is linked to epithelial migration [117,122,123]. The combined use of HCC and laser on scratched keratinocytes led to a reduction TNF-α by 30% more than with laser alone after 24 h. Marked reductions were also observed for gene expression for IL-1α and IL-1β [117].

Research confirmed that the marketed 3.2% HCC enhances the differentiation and proliferation of adipose-derived stem cells after 7 days of treatment, indicating that it could recruit and differentiate stem cells in adipocytes after injection in subdermal fat compartments, thus inducing fat tissue renewal. The cross-linked HA halted cell growth while, from the 14th day, HCC significantly increased proliferation compared to other HAs. The HCC-treated cells demonstrated an increased secretion of the adipogenic biomarkers leptin and adiponectin at 7 days, and this stimulus persisted after 21 days [38]. Furthermore, after adding HCCs to autologous fat grafts employed for lipofilling, an increase in the stromal vascular fraction of adipose stem cells was observed, confirming metabolic viability of adipocytes [124].

Another study confirmed the ability of 4.5% HCCs to sustain the viability of human adipose stromal cells and superiority over H-HA alone. After 21 days of incubation, the highest amount of lipid droplets was observed in HCC-treated samples. It was shown that 4.5% HCCs do not alter the rheological properties of fat, indicating high compatibility with adipose tissue. Furthermore, gene expression analysis confirmed the modulation of relevant biomarkers. Western blotting and enzyme-linked immunosorbent assay (ELISA) techniques confirmed increased secretion of adiponectin and leptin with time [102].

Results of in vitro studies are summarized in Table 1.

Acting as slow-release systems, HCCs exert a synergistic effect of the bio-regenerative role of L-HA and remodeling of H-HA [52]. Taken together, the results of non-clinical studies indicate that HCCs boost the dynamic cell-driven bio-remodeling of extracellular matrix, changing its physical properties through enhancement of viability of keratinocytes, fibroblasts and adipocytes and promotion of extracellular matrix homeostasis and [7,21,27,63]. This effect is superior to natural rejuvenation or biorevitalization, referring to revitalization of skin in a biological way and improving skin nourishment or sole biostimulation (stimulating the skin to self-regenerate), improving the hydration and elasticity of extracellular matrix via an immune-mediated response [63].

## 5. Clinical Evidence

### 5.1. Facial and Neck Rejuvenation

#### 5.1.1. Facial Rejuvenation

Efficacy of 3.2% HCC was evaluated in a 16-week study on 60 women (30 to 60 years of age) in a single center in Italy 4, 8, 12 and 16 weeks after the baseline visit. Product was administered with a 29 G needle at five points on each side of the face, 0.2 mL per point in two sessions 4 weeks apart. Significant reduction in surface microrelief was documented: −16.1% at 4 weeks, −19.4% at 8 weeks, −16.1% at 12 weeks and −12.9% at 16 weeks, translating to reduction of at least 1 grade in the clinical score for 52% of subjects at 4 weeks, 58% at 8 weeks, 55% at 12 weeks and 39% of subjects at 16 weeks. There was significant improvement in reduction of cheek volume loss after 8 weeks (−21.2%) as reduction in clinical score at least by 1 point for 62% of subjects, after 12 weeks (−24.2%) for 70% and after 16 weeks (18.2%) for 55% of subjects. The reduction of wrinkle severity of 11.8% at week 8, 14.7% at week 12 and 14.7% at week 16 corresponded to a decrease of at least 1 grade in clinical score for 35%, 45% and 46% of subjects, respectively. Skin surface hydration improved by 17.1% at 4 weeks, 35.1% at 8 weeks, 39.1% at 12 weeks and 29.4% at 16 weeks post-baseline. From week 8, significant improvement in deep-layer hydration was also observed. According to profilometry analysis on nasolabial folds and marionette lines at week 16, significant reduction was observed for average roughness (−9.7%), total height of roughness profile (−8.2%) and maximum depth (−9.6%). There was also significant decrease in final extensibility (−10%) and viscoelasticity (−17.1%). There was no difference in optical colorimetry parameters. There was an increase of volume of at least 0.2 cm^3^ for 73% of subjects at 8 weeks and 65% of subjects at 16 weeks [27].

A retrospective evaluation on 15 female subjects between 39 and 65 years of age that had undergone Bio Aesthetic Point (BAP) application of 3.2% HCCs 8 weeks apart for treatment of malar and submalar area (1 mL per side during each treatment) showed significant improvement in skin hydration after a single injection (up to 67%), leading up to 87% of improvement in skin hydration after second injection. Significant improvement in viscoelasticity was seen after second treatment. Improvement in skin turgor, brighter skin, reduced nasolabial folds and improved pigmentation and texture were also observed [83].

A study on 11 women 48 to 67 years old showed significant increase in skin hydration one and two months after the first session of 3.2% HCCs. Significant improvement in facial skin elasticity as reduction in Young’s modulus was observed at both evaluation points. There was significant increase in Transepidermal Water Loss (TEWL) both after one and three months. Clinicians rated the product easy to inject for 72.7% subjects and acceptable for 27.3% on a 4-point scale. In the follow-up visits, the effectiveness of the product was rated as optimal in 51.5% of cases, good in 45.5% of cases and satisfactory in 3% of cases on a 5-point scale [84].

A consecutive case series was conducted on 15 women, 38–44 years old, that were injected with 2 mL of 3.2% HCCs in the left face (zygomatic arch, 1 cm medial of the tragus) and neck (at the middle horizontal neck line anterior to the sternocleidomastoid muscle) using a retrograde fanning technique with a comparator on the other side of the face. Moderate-to-pronounced hydration was observed in the first two weeks after treatment for both epidermis and dermis. Three weeks after treatment, there were residual signs of hydration of epidermis and moderate hydration of dermis. The full amount of product could be observed after one week, half amount after two weeks and less than a quarter of amount or none of the product, depending on the point on the face, evaluated after three weeks. After 2 weeks of treatment, up to a 45% increase in mean thickness of dermis could be observed. Immediately after the treatment, more lifting and volumizing effect was observed on the 3.2% HCC side, but this diminished within 2 weeks [37].

A case report of three subjects in which 3.2% HCC was used in two treatment sessions 30 days apart using a blanching technique and a 30 G × 4 mm needle for the treatment of wrinkles, sagging and creases in the upper third of the face reported improvement in skin hydration, density and tone. Reduction in static and dynamic wrinkle retraction of eyelids was observed as a result of improved skin laxity. These results indicate myomodulation properties of the product, as it has been suggested that when injected close to mimetic muscles, the product can cause local mechanical blockade [125,126,127].

Another study on 10 women, 42 to 62 years of age, investigated efficacy of 3.2% HCCs after 3 applications 4 weeks apart. There was evident amelioration of skin roughness and improvement in skin texture. The mean increase in superficial skin hydration 4 weeks after the last procedure at all three measurement points was 29% (*p* < 0.05). According to Cutometer^®^ analysis, there was significant decrease in R0 of 25.1% and F of 47.4%. For reference, higher R0 indicates more stretchable skin, while smaller F0 indicates more elastic skin. According to the Global Aesthetic Improvement Scale (GAIS), score clinicians graded improvement 4/5 (very improved) for 60% of subjects and 3/5 (improved) for 40% of subjects [128].

In a single site-controlled trial, the efficacy of 3.2% HCC was evaluated on 23 (50-to-73-year-old) subjects that received a total of 7 injections each: one at basal visit, one after 4 weeks and then one every 2 months. Follow-up visits were performed 4 weeks after the last injection, 1 year after baseline. All subjects had improvement of at least one grade in the Wrinkle Severity Rating Scale (WSRS) and Facial Volume Loss Scale (FVLS). Significant amelioration was evident from the fifth visit for WSRS and from the third visit for FVLS and was independent of the initial severity in the scale, and was sustained in the 12 months of treatment and follow-up [93].

A case study was published on three women (44, 57 and 59 years old) in which 3.2% HCC was used alongside two other compounds simultaneously as a pre-mixed compound to overcome the rapid diffusion and lack of mechanical resistance to form a hydration reservoir with volume retention. Six months after treatment, all three women had very much improved appearance, as graded by the clinician [2]. Alternating 3.2% HCCs with botulinum toxin was also investigated. In a 64-year-old woman, 3 sessions of 3.2% HCCs, 2 mL per session, in the upper front-lateral area for the forehead and close to the upper portion of temporal crest for temples, 30 days apart, were applied. This was followed with 100 units of Botulinum toxin after another 30 days. At every session there was improvement in skin texture. Distension of the concavities in the forehead and temporal area was also observed. This procedure is not to be advised to subjects with ptosis of the eyebrow tail, ocular bags or a high degree of skeletonization. A 60-year-old woman received repeated injections of 3.2% HCCs ≥ 30 days apart in a combination with a cross-linked filler for treatment of perioral area turgidity and elasticity. In 270 days, the woman received 10 mL of 3.2% HCCs across 5 treatment sessions and 2 mL of cross-linked filler in two separate sessions (after 60 and 210 days). There was positive improvement, as judged by the clinician 4 months from the treatment, with an increase in lip turgidity [6].

A study evaluating efficacy of 3.2% HCCs on Asian skin was published [129]. This is important, as skin differs in different ethnicities. Asian skin is reported to have thicker dermis, increased superficial fat and larger melanosomes compared to Caucasian skin. It is also often more hydrated, with higher levels of collagen and melanin [62,129,130,131]. The study outcomes were reported for 28 women 38 to 60 years of age for 0–4 weeks and 26 women at 12 weeks follow-up. Significant average increase in facial volume was observed just between weeks 4 and 12, while there was a slight decrease in volume at week 12 compared to baseline. According to FVLS and WSRS, there was a decrease in scores over time. Significant increase in superficial skin hydration (21% increase in median value from baseline) was observed for the 6 subjects that underwent this measurement and completed the follow-up. A significant reduction of TEWL (21%) was observed in 26 subjects at week 12 compared to baseline. Furthermore, there was significant reduction in skin tiring or skin fatiguing over time. No difference was observed in skin elasticity measured by Cutometer^®^ [129].

In a study on 10 Chinese women, 30 to 60 years old, living in Italy, undergoing 3.2% HCCs treatment for the face, evaluations were performed at baseline, after 4 weeks at the second injection, and at 8, 12 and at 16 weeks after the first injection (just for 3 subjects due to COVID restrictions). There was significant reduction in WSRS and FVLS clinical scores at week 4 (−22% and −35%), as well as at week 8 (−29% and −48%). There was an increase of 2.6% in superficial skin hydration and 15% increase at 0.5 mm depth hydration at week 4, and 11% and 25% at 0.5 mm depth at week 8. Hydration at 1.5 mm depth increased by 6% at week 4 and 10% at week 8. There was an increase in skin brightness and a decrease in skin redness, and the skin pigmentation doubled [132]. According to comparative analysis results, Chinese women have better and faster improvement in WSRS and FVLS compared to Caucasians. However, researchers did not take the potential influence of subject’s age into consideration [133].

In a single-blinded split face study at UMA clinic in Amsterdam on 24 women of different ethnicities (18 to 65 years old), 3.2% HCC was injected in the right side of the face and evaluations were performed at baseline, after 1 week, 8 weeks and 14 weeks. There was significant increase in surface hydration and TEWL, and significant decrease in pore count, melanin and hemoglobin at 14 weeks on the side where 3.2% HCC was administered [134].

A comparation was done between autologous fat transplantation (5 subjects) and autologous fat transplantation with 3.2% HCCs (4 subjects) two and six weeks after the transplantation for cheek augmentation to assess volumetric durability of the procedures. Nine women, 20 to 57 years of age, completed the randomized controlled pilot study. According to the results, 3.2% HCCs did not significantly increase durability of the transplant at 1 month or 6 months after treatment [1].

More concentrated 4.5% HCC was formulated specifically for restoration of adipose tissue, as it was noticed that the fat distribution in the face changes with aging. On the face, fat compartments may be characterized as superficial (e.g., nasolabial, infraorbital, superficial medial cheek fat) or deep (buccal, medial and lateral e.g., suborbicularis oculi and medial cheek fat). Superficial fat compartments may further be observed as Ghassemi type 1 and 2. Ghassemi type 1 consists of meshwork that coats lobules of fat cells, has weak adhesion to the skin and is located in the posterior of the face. Ghassemi type 2 is composed of dense collagen–muscle fiber meshwork, and is found medial to the nasolabial fold. With age, either ptosis appears (saggers) or downward migration coupled by a decrease in fat of lateral cheek compartment of the face and a decrease in mean size of adypocites in the deep fat alongside hypertrophy of the middle cheek compartment (sinkers). Hence, adipose tissue is a reasonable target in aesthetic medicine. In subjects presenting with ptosis, the so-called saggers, application of the 4.5% HCCs using a 25 G × 38 mm cannula to the zygomatic (0.5 mL) and preauricular (0.5 mL) area is advised for restoration of the fat shift in the middle cheek and lifting effect. Preauricular injection technique for the restoration of fat loss in the lateral cheek is preferred for the so-called sinkers. Injection of 1 mL of 4.5% HCCs using a 22 G × 50 mm cannula is recommended [95,98,99,135,136,137,138,139,140,141,142,143].

A study was conducted on 50 women, 40 to 70 years old, to evaluate 4.5% HCCs’ efficacy in restoring the face volume. The product was administered to the lateral cheek fat compartment, specifically the superficial fat compartment of the preauricular area, using a 25 G × 50 mm cannula in a single entry point 2 cm from the tragus utilizing retrograde injection (1 passage, 1 mL). Treatment was administered in two sessions 30 days apart, and a follow-up visit was 3 months after the second session. There was significant reduction in FVLS and WSRS at 30 days that was sustained 3 months after the second treatment session (*p*  <  0.05). Reduction of at least 1 grade in FVLS was observed in 36% of the subjects after 30 days and in 64% of subjects 3 months later. Reduction of at least 1 grade in WSRS was observed in 38% of the subjects after 30 days and in 51% of subjects 3 months later. The clinicians rated the product with good tolerance in 89% of cases and excellent tolerance in 11% of cases [99].

Furthermore, according to a retrospective case series, this product was administered to the superficial fat compartment along the line from the preauricular area to the mandibular angle using a 25 G cannula in the subcutis at 3–4 mm depth to 22 subjects, 36 to 60 years of age. There was significant increase in skin thickness one and three months after the first treatment. Moreover, an increase of the injected fat compartment was confirmed after one month. Six months after treatment, there was insignificant reduction in thickness, but results were still visible [100]. Table 2 outlines characteristics of the mentioned studies.

#### 5.1.2. Neck Rejuvenation

In a study that included 23 women, 41 to 65 years of age, treated for neck skin laxity with evaluations after 1 and 4 months from the first injection, improvement in the neck skin laxity score of at least 1 was evident in more of the half of studied population. There was reduction in skin laxity and roughness of 15% at 1 month with improvement in 48% of subjects and 21% reduction in skin laxity and roughness at 4 months with improvement in 65% of subjects. In instrumental evaluation there was significant 22% reduction in average roughness at 1 month and 24% at 4 months, 19.4% reduction in total height of roughness profile at 1 month and 22.2% reduction at 4 months and 22% reduction in maximum depth at 1 month that sustained to 4 months. Furthermore, significant increase in superficial skin hydration was observed at 4 months (22.6%), in deep skin hydration measured at 0.5 mm at 1 month (6.1%) and in deep skin hydration measured at 1.5 mm depth at 4 months (8.4%). In plastoelasticity measurements, significant increase was observed for immediate elastic recovery at 4 months (increase of 14.2%) [59].

Efficacy of 3.2% HCCs on facial skin rejuvenation was evaluated on 9 women and 1 man (26 to 62 years of age) belonging to Central Eastern European ethnic subpopulation with Oriental mongoloid features. The BAP technique of injection was compared to diffuse injections in two sessions 4 weeks apart. There was significant improvement in skin hydration. Improvement in skin elasticity and amelioration in melanin was present but not significant. There was significant decrease in mean roughness of 9.2%, 8.5% size decrease in wrinkles and 4.5% average decrease in skin pigmentation [66].

In a study on Chinese women, 30 to 60 years old, living in Italy, 18 women underwent 3.2% HCCs treatment for neck laxity utilizing the 10-point BAP technique and were evaluated at baseline, after 4 weeks at second injection, at 8 weeks and 12 weeks after the first injection. The neck skin laxity clinical score reduced by 33% at week 4 and 45% by week 8. There was an increase of 11% in superficial skin hydration and a 6% increase of hydration at 0.5 mm depth at week 4, and 25% in superficial and 13% at 0.5 mm depth hydration at week 8. Hydration at 1.5 mm depth increased by 2% at week 4 and 7% at week 8. There was an increase in skin brightness and a decrease in skin redness, and the skin pigmentation doubled [132]. According to comparative analysis results, Chinese women have better and faster response to the product according to neck skin laxity clinical score compared to Caucasians [133].

A real-world data study included records of 10 clinicians from Indonesia (*n* = 4), Singapore (*n* = 3) and Malaysia (*n* = 3), treating subjects’ neck laxity with 3.2% HCC using a 29 G needle and the BAP technique followed by a light massage to ensure distribution of the product. Treatment was applied at baseline and after 30 days. Follow-up was after 90 days from baseline. There were a total of 26 subjects, 31 to 61 years old, evaluated, of which 5 were men. There was a reduction in the mean neck skin laxity score from 3.08 at baseline to 2.62 after 30 days and 2.12 after 90 days (*p* < 0.001). According to a clinician’s interpretation, there was much improvement in neck skin laxity after 90 days (4/5), and improvement (3/5) in skin texture and augmenting skin firmness or tone [21].

Another similar real-world data study on 14 Japanese women (53–75 years old) had a baseline mean neck skin laxity score of 3.0 with significant reduction at 30 days to 2.29 and at 90 days to 1.93. There was improvement in skin laxity after 30 days for 9 subjects and much improvement for another 3 subjects. All subjects had improvement of at least one grade by 90 days of follow-up. At 90 days follow-up, improvement (3/5) in skin laxity was seen in 9 subjects and much improvement (4/5) it the remaining 5 subjects. There was improvement (3/5) in skin firmness after 30 days for 7 subjects and much improvement (4/5) for another 2 subjects. At 90 days follow-up, improvement (3/5) was seen in skin firmness in 11 subjects, much improvement (4/5) it another 2 subjects and no improvement (2/5) in 1 subject. Improvement (3/5) in skin texture was evident in 12 subjects at 30 days, and 13 subjects at 90 days, with one showing much improvement (4/5) [144].

Ten women (35 to 65 years old) were treated with 3.2% HCC and plasma exeresis for neck skin laxity. Treatment was administered in two sessions 30 days apart and follow-up visit occurred 2 months after the initial treatment. Clinical improvement was visible in all subjects, with 1 having very much improved, 6 much improved and 1 improved. Subjects with more pronounced signs of aging had greater improvement. These results persisted for 6 months [145]. For treatment of the neck in a 63-year-old woman and a 56-year-old woman, 50 IU of microbotox were administered followed by 3.2% HCCs after 15 days and 45 days, 2 mL at each session. A clinician noticed an increase in skin elasticity and reduction in skin laxity [6].

### 5.2. Body Applications

A study on 10 women with skin laxity and roughness on the dorsal side of the hands 35–55 years of age evaluated 3.2% HCCs. Treatment was applied in two sessions 4 weeks apart, 1.5 mL of product was administered to each hand using 22 G × 50 mm microcannula, single entry point, fanning technique with 5 passages, 0.3 mL per passage. On a 5-point scale, with 1 being much improved, a plastic surgeon and a dermatologist reported 2.1 ± 0.7 improvement on the left hand and 2.2 ± 0.6 of improvement on the right hand after one month and 1.5 ± 0.7 on the left and 1.6 ± 0.7 on the right hand 4 months after treatment. Significant increase in skin thickness (epidermis with dermis) was observed over time, as assessed with ultrasound (7.6 ± 1.1 mm at baseline, 7.9 ± 1.5 mm after one month and 9.4 ± 1.6 mm after 4 months, *p* < 0.001). Similar results were observed for total thickness of skin with subcutaneous layer (31.9 ± 5.3 mm vs. 33.10 ± 6.2 mm vs. 34.7 ± 5.5 mm), indicating improvement over time [146]. A similar single-center study on 46 subjects (38 to 65 years of age) demonstrated significant improvement in skin laxity 4 months post-start of the treatment and in the resistance to pinching clinical score after both one and four months (*p* < 0.05). There was significant reduction in skin roughness after 4 months (17 μm, 13%) and significant reduction in wrinkle maximum depth after one month (31 μm, 12.2%). After four months, there was significant improvement in immediate extensibility, viscoelasticity and immediate elastic recovery as assessed with a dermal torque meter. For final extensibility, significance could not be reached [147].

A single-center study evaluated 3.2% HCC body formulation for treatment of inner arm and abdomen laxity (grade 3–4/5 with 5 being very severe laxity) in 22 women (47 to 65 years). The Bio Aesthetic Point (BAP) technique of 10 micro wheals at 3 horizontal levels was used to avoid large vessels and nerve branches to minimize risks and mistakes and maximize product diffusion. Inner arm laxity reduced by 12% after 1 month (reduction of 1 grade in 45% of participants) and by 29% (reduction of 1 grade in 86% of participants) after 4 months from the start of the treatment. For abdomen laxity, significant reduction was observed at the second assessment point as 23% reduction in laxity, with reduction of visual score in 68% of participants. At the second assessment point, there was 27.2% reduction in skin roughness, 21.6% improvement in the total height of roughness profile and 18.8% improvement in maximum depth. Significant improvement in skin hydration was observed at both assessment points. In inner arm, there was 17.1% and 15.6% improvement, and in abdomen, 16.1% and 11.1% improvement in surface hydration. Significant improvement was observed for deep skin layer hydration inner arm skin 4 weeks after start of the treatment. There was a 10.6% improvement in skin hydration at 0.5 mm depth and 11.7% improvement at 1.5 mm depth. For abdomen, significant improvement was observed for skin hydration at 1.5 mm depth by 13.7% at the first assessment point and 9.2% at the second assessment point. In evaluation of skin plastoelasticity, significant improvement was observed for inner arm final extensibility at the first assessment point (−10.8%) and in immediate elastic recovery at both the first and second assessment points (−24.0% and −26.6%). For abdomen, significant reduction and an immediate elastic recovery of 24.0% was observed 4 months after first injection [148]. Furthermore, 3.2% HCC was administered for inner arm laxity to a 72-year-old woman who could not have surgery. There were 3 sessions 30 days apart, and 2 mL was injected in each arm at every session. A clinician rated improvement with 7–8 points, slightly higher than the subject [6].

In another single-center study on 50 women (35–65 years of age), using the BAP technique, 3.2% HCC body formulation in two treatment sessions 4 weeks apart was used to treat mild skin roughness and laxity on inner arm, abdomen and knees. There was significant improvement in skin laxity across all treated areas one and four months after the beginning of the treatment (*p* ˂ 0.05). Skin hydration at 0.5 mm depth was markedly improved at both assessment points for inner arm and abdomen, and one month post-treatment for knees. These results could not be replicated for skin hydration at 1.5 mm depth, as only significant improvement was seen at the second assessment point for inner arm. Significant improvement in mean roughness was measured for inner arm and abdomen at both assessment points, and for knees four months after start of the treatment. Significant improvement of maximum depth of the skin profile was measured for inner arm at both assessment points and for abdomen at the second assessment point. No significance was observed in the measurements of this profilometric parameter for the knees [96].

Interestingly, 3.2% HCC body formulation was administered in one arm of 9 ex-obese women (35–50 years old) experiencing skin laxity in two sessions 30 days apart. One month after the second treatment, samples were taken for histological analysis when subjects were operated and compared to the samples of the contralateral not-treated arm. Significant increase in elastic fibers and more regular deposition and architecture of elastin, as well as signs of bioactivation of fibroblasts, were observed [149].

### 5.3. Acne Scars and Other Indications

#### 5.3.1. Ane Scars

In treatment of acne scars, adding 3.2% HCCs (1 mL) with a 29-gauge needle to subcision was compared to subcision alone. In a single-blinded, split-face randomized controlled trial on 3 men and 9 women, 19–42 years of age, and Fitzpatrick skin type II-IV. On the intervention side, first the fibrous tissue under the scar was released by moving the 29-gauge needle back and forth; then, 3.2% HCC was added alongside lidocaine 2% and epinephrine (1/100) to prevent hematoma, and then the final subcision was performed with a 25-gauge cannula to ensure distribution of the product. The same procedure was repeated after one month. Assessments were performed at baseline and 3 months after the final procedure. No significance was observed in the mean growth rate of sonographic depth (*p* = 0.424) between the two sides, although there was a 29.74% increase in the mean growth rate on the treatment side compared to 22.27% on the subcision-alone side. There was no significant difference based on the global improvement scale (*p* = 0.890), image analysis or clinical scoring. The rolling subtype of acne scars had the best response to both treatment arms, slightly higher on the intervention side but with no significance between them. Moreover, the boxcar subtype had significant decrease in clinical score on the subcision side (*p* = 0.007) [52].

A similar study included 12 subjects 23 to 52 years of age, with Fitzpatrick skin type II-IV with follow-up after one, three and six months. Scars were treated with 0.02–0.1 mL of 3.2% HCCs, and after the subcision areas were massaged to flatten the product and ensure correct placement. A dermatologist’s mean global evaluation score 1 month after treatment was 3 ± 0.5. After 3 months, moderate improvement was observed (2.5 ± 0.49), and these results were persistent after 6 months (2.5 ± 0.43) [4,50].

In a different study on 82 randomized subjects (19 to 33 years old) a triple-step acne scar revision technique (TSASRT) was evaluated 6 months after treatment. First, the fibrous cords were cut with a NOKOR needle 18 G in fanning direction, after which 3.2% HCC (0.02–0.1 mL) was injected with a 29 G needle in the scar’s anthropic dermal component, and the third step involved filling subcised space with 0.02–0.1 mL of 3.2% HCCs using 25 G cannula. After 6 months, there was significantly greater acne scar reduction for rolling and boxcar scars, shorter duration of bruising and less scar reappearance in the treatment arm. At least 70% improvement, as observed by both investigators and subjects, was significantly more frequent in the treatment arm, translating to significantly greater mean improvement on a 12-point Goodman and Baron grading [4,150]. It is suggested that HCCs occupy space after incision—thus preventing fibrous bands from forming again, create ideal media for collagen formation, enhance scar healing and reduce the number of subcisions needed [150].

One study evaluating dual treatment with 0.02 to 0.1 mL of 4.5% HCCs and including 38 subjects (20–68 years old), Fitzpatrick I-III, among which 25 females reported 4-month treatment outcomes as follows: significant decrease of 20.4% in scar deepness (*p* < 0.001) measured as mean profilometry, and 30.8% improvement in mean acne scar Goodman and Baron grade (*p* < 0.001); by clinical evaluation, 32% of subjects had significant improvement, 36% improved, and in 11% there was no change [51].

A double-blind randomized study comparing cross-linked HA with 3.2% HCCs concluded that cross-linked HA achieves faster results, but with HCC there is improvement at later visits during 6 months follow-up based on investigator global assessment (IGA) [4,73].

Furthermore, a combination of non-ablative laser and HCC was used in treatment of acne scars. The laser was used in 4 consecutive treatment sessions every 4 weeks and complemented with 3.2% HCCs immediately after the first and the third sessions. A total of 12 subjects, Fitzpatrick I-III, 32 to 57 years old were evaluated at baseline, and one and three months after treatment. Mild-to-moderate improvement was recorded for all subjects [47].

#### 5.3.2. Other Skin Conditions

Since there is an increased expression of CD44 and reduced distribution of HA in inflamed psoriatic skin, HCC was evaluated in treatment of psoriasis plaques (two women 37 and 55 years old, one man 45 years old). For this purpose, 0.2 mL/2 cm^2^ of treated area of HCC was injected to three patients in two intervals not shorter than 4 weeks. There was improvement in skin texture, reduction in redness, scaling, desquamation and pruritus [151]. In another clinical case series, the efficacy of 3.2% HCCs was investigated in 6 patients for treatment of psoriasis. Intra and peri lesional application of the product was conducted in two sessions 4 weeks apart. There was reduction of pruritus from mean 7.0/10 at baseline to 2.0 after 4 weeks and 2.2 after another 4 weeks. The Psoriasis Area and Severity Index reduced from 4.4 to 2.1 at the first assessment and 1.8 at the second assessment. This was evident in reduction of desquamation, scaling and redness. Five of six patients had significant improvements, while one improved gradually up to week 3 post-treatment but later worsened, with no reduction in PASI onwards and with reappearance of pruritus at the second assessment point [152].

When administered with 3.2% HCCs for facial features, one subject suffering from occasional acne vulgaris reported significant decrease in rash after second treatment and overall reduction in the severity of post-acne stains. Another subject had remission of peeling in wintertime alongside less redness of the skin [66].

It was suggested that the physical tension on the extracellular matrix related to jet-induced delivery of HA results in micro-injury to collagen fibers and initiates the healing mechanism with no excessive scarring. It is believed that the jet-induced neocollagenesis is not the result of inflammatory response, as the tears in the dermal matrix do not reach the threshold for triggering inflammatory response. It is assumed that the process is similar to noninflammatory healing. The delivered HA delays the senescence of mesenchymal cells boosting this effect, as senescent mesenchymal cells are usually related to scarring [43,114]. When a postsurgical abdominal hypertrophic scar was treated with multiple jet injections of 3.2% HCCs, there was release of skin tension and flattening of the scar observed 6 months after treatment, as well as relief of associated discomfort and pain [43]. In another case, a 54-year-old female was treated for a burn scar contracture with jet-injected HCCs diluted to 3.5 mg/mL. After 3 months, the scar was more palpable and softer, and 18 months after single treatment, there was improvement in scar appearance; the scar had softened and was visually flatter with normalization in skin color. Full range of motion of the affected joints was restored. The Vancouver Scar Scale score decreased from 9 to 3. A 36-year-old man was treated with three monthly sessions of jet-injected HCCs for post-surgical scarring restricting movement with persisting pruritus. Pruritus subsided after the first treatment; there was progressive softening and flattening of the scar by the third month, and at follow-up 6 months from the last treatment, there was increase in motion. The Vancouver Scar Scale score decreased from 11 to 6 [153].

### 5.4. Subject Satisfaction

Efficacy of 3.2% HCCs was evaluated on over 60 women a 16-week study. Marked improvement in skin smoothens was observed by 48% of participants, and medium in 32%, while 38% experienced marked improvement in skin firmness and 46% medium improvement. Around a third (34%) experienced marked improvement in skin hydration, 4% reported very marked improvement in this parameter and 48% medium improvement. Skin brightness improved very markedly for 4% of participants, markedly for 28%, while medium improvement was observed in 45% of subjects. Medium improvement in lifting effect was observed by half of the participants, with additional 18% stating the improvement was marked and another 2% noticing very marked improvement. Medium reduction of deep wrinkles was noticed in 55% of subjects, marked in additional 14% and very marked in 4%. Very marked reduction in superficial wrinkles was observed by 4%, marked by 30% and medium by 45% of subjects. Least pronounced was the reshaping of face silhouette, with 21% of participants experiencing marked improvement and 43% medium improvement [27]. In a retrospective evaluation of 15 female subjects undergoing 3.2% HCCs treatment for the face, two subjects were unsatisfied after first treatment, ten were satisfied and three very satisfied. After the second treatment, nine were satisfied and six were very satisfied [83]. In a study on 30 women (40 to 68 years of age) who received treatment with 3.2% HCCs 30 days apart, 13 saw 76–100% improvement at 30 days, and another 13 saw 51–75% improvement. After 60 days, 22 saw 76–100% improvement, with 8 seeing 51–75% of improvement in facial aesthetic outcomes [154]. Furthermore, in a study on 11 women, 48 to 67 years old, that received treatment with 3.2% HCCs for their facial features, 87.9% of them were very satisfied and 12.1% were satisfied with the result as rated on a 3-point scale [84].

In a study on 10 women, 42 to 62 years of age, in which 3.2% HCCs was administered 3 times 4 weeks apart, 60% of women rated their appearance as very improved (4/5) and 40% (3/5 as improved according to GAIS [128]. A trial was conducted in which 3.2% HCCs was evaluated on 23 subjects (50 to 73 years old) that received a total of 7 injections each, one at the basal visit, another after 4 weeks and then one every 2 months. Follow-up visits were performed 4 weeks after the last injection and 1 year after baseline, and 95% reported amelioration for WSRS, 99% for FVLS [93]. In a case report of three subjects in which 3.2% HCC was used in the upper third of the face, all three experienced exceptional improvement on GAIS 6 months after treatment [125].

The three women which had undergone a procedure with 3.2% HCCs pre-mixed with two other compounds all reported very much improvement in their appearance [2]. In a split-face study on 24 women in which 3.2% HCCs was injected in the right side of the face, overall, subject satisfaction score had an increase of 22.2% (*p* < 0.001). Significant increase was observed for subject satisfaction in terms of hydration (21.8%), elasticity (24.9%), skin texture (20.0%), skin thickness (14.8%) and fine line reduction (20.6%) [134]. In another study comparing 3.2% HCCs on the left side of face and neck with another product on the other side, subjects noticed a sustained improvement in skin hydration, firmness, elasticity and complexion. Furthermore, greater improvement according to GAIS was recorded on the 3.2% HCC side after 3 weeks, indicating the bioremodeling effect, as 3.2% HCCs could not be detected at this time point [37].

When autologous fat transplantation (5 subjects) and autologous fat transplantation with 3.2% HCCs 2 and 6 weeks after the transplantation (4 subjects) for cheek augmentation to assess volumetric durability of the procedures were compared, subjects in the 3.2% HCC group reported higher GAIS scores at one month of follow-up, indicating significant early improvement in aesthetic outcome; however, there was no significant difference between the two groups at 6 months follow-up [1].

In a study on 50 women, 40 to 70 years old, evaluating 4.5% HCCs’ efficacy in restoring the face volume, 34% of women saw very marked reduction of deep wrinkles, 4% marked 46% medium and 16% light on a 4-point scale. Reduction in superficial wrinkles was very marked for 24%, marked for 6%, medium for 44% and light for 26%. Very marked improvement of skin firmness was noticed by 4%, marked by 50% and medium by 46%. Very marked improvement of skin smoothness was reported by 2%, marked by another 2% and medium by 56%. Marked improvement in lifting effect was observed by 8%, medium by 52%. Marked improvement in skin brightness was noticed by 6%, medium by 54%. Marked improvement in skin hydration was observed by 2%, medium by 54%. Marked reshaping of face silhouette was observed by 6%, medium by 50%. Other women reported light amelioration in the mentioned skin parameters as none stated that the improvement was absent. Three months after the second procedure, 68% of subjects marked the procedure with excellent tolerance and another 24% with good tolerance [99].

After being injected with 4.5% HCCs in the superficial fat compartment of the face, 77% of 22 subjects reported improvement one month after the first treatment session and 91% one month after the second treatment session. At one month post-treatment, 18% marked results as very improved, while this was stated by 4% of subjects one month after the second session. There was no report of worsening at any of the evaluation points [100].

In a study evaluating efficacy of 3.2% HCCs on Asian skin, 26 women rated the tolerance of the product after 4 weeks as average 4.36 on a 5-point scale and as 4.00 after 12 weeks of follow-up [129].

Adding 3.2% HCCs to subcision of acne scars significantly increased subject satisfaction, with one study reporting an increase in VAS (10-point visual analogue scale) score for patient satisfaction from 26.58 to 69.25 compared to 39.83 to 63 on the subcision-alone side (*p* = 0.021) [52]. In a similar study evaluating a dual-plane method for acne scars, six months after treatment, 8 out of 12 subjects reported moderate improvement, 2 marked improvement and 2 minimal improvement [50]. When TSASRT was employed, 39/42 subjects reported an A grading compared to 19/40 in the control group [150]. In a study on 12 subjects combining non-ablative laser and 3.2% HCCs in treatment of acne scars, subjects reported satisfaction of 3–5 on a 5-point scale after 3 months of follow-up [47].

In a study that included 26 subjects from Indonesia, Singapore and Malaysia treated for neck skin laxity, subjects reported improved-to-much improved skin laxity and augmenting of skin firmness or tone (3–4/5) after 90 days. They reported very much improvement (5/5) in skin texture after 90 days. There was high level of satisfaction with treatment outcomes [21]. In another similar real-world data study on 14 Japanese women (53–75 years old), 8 observed improvement in skin laxity after 30 days and 4 much improvement. At 90 days follow-up, improvement was seen by 7 subjects and much improvement by 6 subjects, while one dropped from improved at 30 days to no improvement at 90 days. There was improvement in skin firmness after 30 days observed by 8 subjects and much improvement in another 3 subjects. At 90 days follow-up, improvement was seen by 5 subjects, much improvement by another 7 subjects and no improvement by 2 subjects. Improvement in skin texture was evident for 7 subjects at 30 days, and 8 subjects at 90 days, with 6 claiming much improvement. All subjects stated they would recommend the treatment [144]. In another study in which 23 women 41 to 65 years of age were treated for neck skin laxity, 87% saw medium-to-very marked improvement in skin roughness, 92% in skin laxity, 91% found the silhouette remodeled and more defined, 96% lifting effect, skin suppleness and skin hydration and 91% saw improvement in skin smoothness [59].

When 3.2% HCCs was combined with cross-linked HA filler, for treatment of perioral area subject reported a high degree of satisfaction with the treatment result. In combination of 3.2% HCCs with botulinum toxin for neck laxity, subjects graded satisfaction with 8 points, and for forehead treatment, the subject was very satisfied and willing to repeat the treatment [6].

From ten women (35 to 65 years old) that were treated with 3.2% HCCs and plasma exeresis for neck skin laxity, 4 observed very much improvement 2 months after the initial treatment, 5 much improvement and 1 improvement according to GAIS [145].

Subjects with skin laxity, limiter muscular ptosis and adipose tissue may be good candidates for 3.2% HCC body kit. A multi-center assessment of real-world data on subject satisfaction after application of injections and 3.2% HCCs body patch on days 0 and 30 for three to five hours with application of 3.2% HCCs body cream every day in between was conducted in centers in Netherlands (*n* = 15) and Italy (*n* = 35). The majority of the treated subjects were women (85%), 40 to 64 years old. Skin laxity on décolleté (20%), inner arms (35%), abdomen (35%) or knees (10%) was treated using the Bio-Aesthetic Points (BAP) technique developed by the manufacturer for 3.2% HCCs, 10 injection points across 3 horizontal levels in a 3–4–3 pattern. Great subject adherence was observed, 97% for the patch and 95% for the cream. Almost half of the subjects (48%) reported that the kit improved skin hydration very much, and an additional 28% stated that there was much improvement in skin hydration on a 4-point scale. For skin laxity 62% reported a lot of improvement and 18% very much improvement. For skin tone/firmness, 36% reported much improvement and 22% very much improvement. No improvement was observed for skin laxity and skin tone/firmness in 4% of subjects and for skin hydration in 2% of subjects. More than half of the subjects (56%) reported that the kit enhanced their body and appearance, an additional 12% said there was very much improvement and 10% did not notice any improvement. Much improvement was seen in social relationships for 42% of subjects, very much for 6% and not at all for 16% [35]. Subjects may have higher-than-realistic expectations. In one study evaluating the effect of HCC on skin laxity of the hands, marked differences were observed between the grading provided by health care professionals and subjects, as the greatest improvement was observed by a plastic surgeon, then by a dermatologist and lastly by subjects [146]. However, in three separate studies in which subjects were treated for neck laxity, they reported higher gradings for improvement compared to clinicians. It should be noted that two of the mentioned studies were conducted on an Asian population [21,144,145]. Moreover, a 72-year-old woman who could not have surgery was treated for inner arm laxity with 3.2% HCC. There were 3 sessions 30 days apart, and 2 mL was injected in each arm at every session. The subject rated improvement with 6–7 points, slightly lower than the clinician [6].

In a study evaluating 3.2% HCC body formulation for treatment of skin laxity and roughness of inner arm, abdomen and knees, all 50 women reported marked improvement in skin laxity and roughness and less improvement in skin hydration after the two-session treatment [96]. When 22 women were treated for inner arm and abdomen laxity with 3.2% HCC body formulation, they observed the greatest improvement in skin smoothness of the inner arm (81.8%), followed by improvement in skin roughness and skin hydration of the inner arm (77%) and then skin laxity and suppleness, as well as more defined and remodeled silhouette (68%). The least grades were given for the lifting effect of inner arm and definition of abdomen silhouette (63.6%). Furthermore, for abdomen, the greatest improvement was seen in skin suppleness (73%), followed by skin smoothness and hydration (68%) and then skin roughness, laxity and lifting effect (64%) [148]. When 3.2% HCC body formulation was used on 46 persons for treatment of skin laxity and roughness on hands, subjects gave positive feedback related to the treatment effectiveness, antiwrinkle properties and the transparency of skin [147].

The 42-year old woman with atopic dermatitis symptoms persistent on the face that was treated with HCC was very satisfied with the results two years after the first treatment, and there was no evidence of relapse [155].

## 6. Safety and Tolerability

For the administration of HCCs, a special technique was developed to minimize safety risks. The BAP technique involves identifying 5 injection spots with less chance of hematoma and bruising, ultimately resulting in fewer treatment sessions. For the face, these points are:

- at the maximum projection of the zygomatic arch, minimum 2 cm from the lateral canthus of the eye;
- 1.5 cm anterior to the inferior margin of tragus;
- 1.5 cm above the mandibular angle;
- 1.5 cm toward the labial commissure from the intersection point between a vertical line in the middle of the chin and a perpendicular line drawn at a third of that line;
- at the node wing 1.5 cm from the nasal base at the intersection point between the perpendicular line from the pupil midline and the horizontal line starting from the nasal base to the tragus [83,129].

Some authors have expressed concerns relating to the medial cheek injection point lateral to the nasolabial fold, as vascular complications may occur due to facial arteries [134]. The specific formulation allows for delivery of high HA concentration with a 29 G thin wall needle slowly that leads to reduced pain, as injections are slower and there is greater subject compliance [83].

In a mixed methods study that synthesized global data including 2.28 million social media posts, 37,250 media articles and 457 peer-reviewed studies, 3.2% HCCs exhibited the lowest hazard ratio for complications among the investigated non-surgical aesthetic treatments, as well as the lowest severe complication rate of 0.3%. Significant reduction in complication rates was observed after the introduction of 3.2% HCCs, as the average complication rate for skin boosters dropped from 5% per month to 2.3% per month. The adjusted complication rate for HCCs was the lowest (2.1%) as well, and the calculated probability of safety the highest, at 90% [24].

A study analyzing post-marketing data 3 years after marketing, and more than 40 thousand subjects treated, collected data from February 2015 to February 2018. In this period, 12 reports were collected by the manufacturer from the first, third and fourth country by sales. In 3 reports, the product was not considered related to adverse events. None of the events were serious, and all were attributed to improper injection technique rather than the product. Edema, redness, swelling, ecchymosis and erythema were reported alongside sensitization after the second treatment session [85]. An update was published evaluating post-marketing adverse events for 3.2% HCCs from January 2018 to October 2023, with an estimated 1,091,956 subjects exposed, and for 3.2% HCC body formulation, from January 2020 to October 2023, with an estimated 27,692 subjects exposed. Adverse events occurred in not more than 0.04% of exposed subjects for both products. Injection site conditions (edema, erythema, nodule, pain, swelling) and skin conditions (dry skin, erythema, pruritus, rash, skin wrinkling) were the most reported for 3.2% HCCs. There was a single case of vascular adverse event (embolism or vascular occlusion). In the given analysis, it is likely that the number of exposed subjects was underestimated, as they were calculated on the basis number of sold syringes divided by 7, due to the fact that a single person can receive treatment twice in 30 days and then every 2 months for maintenance, so the maximum is 7 per year per subject. It is highly unlikely that every person received 7 treatments in a year. For 4.5% HCCs, there were 11 reports of adverse events, mostly administration site conditions (injection site hypersensitivity, inflammation and edema) and subcutaneous tissue disorders (erythema, idiopathic guttate hypomelanosis, pruritus, skin hyperpigmentation). Despite the low overall risk of adverse events and complications with 3.2% HCCs for an individual person, as demand increases and more and more subjects are exposed to the product, and more product is used, there will be an increase in the number of complications and adverse events; hence, the greater risk for the society. For reference, there was a notable increase in the estimated proportion of exposed subjects with a safety complaint for 4.5% HCCs from 2022 (0.002%) to 2023 (0.013%) [106]. Minimization of adverse events can be achieved by injecting 4.5% HCCs with a cannula implementing retrograde injection. The use of a cannula lowers the odds of occlusion by 77.1% compared to injection using needles. For the periauricular area, a 22 G × 50 mm cannula is recommended, and an entry point 2 cm from the tragus. For both the preauricular and zygomatic areas, a 25 G × 38 mm cannula is recommended, and an entry point 3 cm from the tragus [95,156].

Long-term safety of 3.2% HCCs was evaluated in a single site-controlled trial that included 23 (50 to 73 years old) subjects. The subjects received injections at the basal visit, after 4 weeks and then every 2 months for a total of 7 injections. Follow-up visits were performed 4 weeks after the last injection and 1 year after baseline. There were no adverse events in 17.4% of subjects, while 65.2% had ecchymosis lasting 5 to 7 days. Another 17.4% had small bumps lasting 7 to 10 days. The mentioned adverse events were related to the injection procedure and not to the product itself [93]. In a 16-week study on 60 women injected with 3.2% HCCs, 9 subjects had light bruises after the first and second treatments and another 5 had light edema that disappeared 3 to 10 days after injections. One subject reported light pinching sensations after first session that disappeared within two weeks. After the second treatment, the subjects rated the product’s tolerability as excellent in 52% of cases and as good in 48% of cases. The clinicians rated it as excellent in 70% of cases and good in 30% of cases, indicating slightly higher sensitivity in subjects undergoing the treatment [27]. Furthermore, in a retrospective evaluation of 15 female subjects undergoing 3.2% HCCs treatment for facial rejuvenation, there was reported bruising in two treatments and persistent swelling in one case. All adverse events subsided within two days [83]. In another study on 30 women (40 to 68 years of age) who received treatment with 3.2% HCCs in two separate sessions, there were 3 cases of ecchymosis, and 2 women complained of pain [154]. In a study on 11 women that received treatment with 3.2% HCCs for their facial features, 12.1% had localized hematomas that dissolved within 2 to 3 days [84]. In a split-face study on 24 women in which 3.2% HCC was injected in the right side of the face, skin irritation was reported by 7 women, hematoma by 6, nodules by 4. All adverse events were mild and resolved in a few days without intervention [134]. In a consecutive case series on 15 women 38–44 years old, all subjects had light pain 2 to 3 days post-procedure and one reported bruising [37].

A case report of three subjects in which 3.2% HCC was used in the upper third of the face, there was slight edema in lower eyelids and localized hematoma that subsided within 2–7 days [125].

When 3.2% HCC was combined with cross-linked HA filler for treatment of perioral area, the subject reported mild discomfort due to swelling immediately after injections that disappeared within a few hours alongside rare bruising. In combination of 3.2% HCCs with botulinum toxin for neck laxity, small wheals appeared at the injection sites but subsided within 24 h, and for forehead treatment, the subject reported mild pain [6]. In a case study series on three women using pre-mixed 3.2% HCC with two other compounds, no adverse events were reported during 6 months of follow-up [2].

In a pilot study combining autologous fat transplantation with 3.2% HCCs for cheek augmentation on a total of 9 women, only self-limiting mild erythema and localized swelling were noticed, and these reactions resolved within a few days with no treatment [1].

In a study on 50 women, 40 to 70 years old, evaluating 4.5% HCCs’ efficacy in restoring the face volume, light bruises appeared in 8% of women and resolved within 5 to 10 days [99].

In a split-face study of adding 3.2% HCCs to subcision in treatment of acne scars on 12 subjects the occurrence of erythema, swelling and significant pain was the same for both sides, while ecchymosis occurred in 9% of cases on the treatment side, compared to 100% on the subcision-alone side. There was a single case of post-inflammatory hyperpigmentation on the subcision alone side (9%). All adverse events were mild and transient in nature [52]. In a similar study evaluating dual-plane method for acne scars on 12 subjects, the mean pain score on a 10-point visual analogue (VAS) scale was 2.3 ± 1.3 after the first and 3.3 ± 0.9 after the second session. Two subjects had visible deposits after injections that diminished spontaneously with no treatment [50].

In a study investigating TSART with 42 subjects in treatment arm, edema diminished within 2–5 days, bruising diminished within 7 to 10 days, and there was no hypertrophic scars, hyperpigmentation, hemorrhagic papule or pustule [150]. In a study using 4.5% HCCs for dual treatment of acne scars on 20 subjects, no adverse events at 6 months follow-up [51].

In a study on 12 subjects combining non-ablative laser and 3.2% HCCs in treatment of acne scars, subjects reported moderate pain at 3.8 ± 1.5 on VAS scale, all adverse events were mild and well-tolerated, including transient erythema and edema. At 3 months follow-up, no additional adverse events were reported [47].

In a study that included 26 subjects from Indonesia, Singapore and Malaysia treated for neck skin laxity, VAS pain scores were 2/10 after the first injection and 2.2/10 after the second injection [21]. In another similar real-world data study on 14 Japanese women (53–75 years old), the average VAS pain score after the first injection was 2.36, and 2.5 after the second injection [144]. A satisfaction survey was developed to investigate pre- and post- treatment of the neck using hybrid cooperative complexes of hyaluronans in a cohort of Japanese patients. In another study in which 23 women 41 to 65 years of age were treated for neck skin laxity, light bruises appeared in around half of them and disappeared in a couple of days. The procedure was rated with excellent tolerability by 83% of women and with good tolerability by the remaining women [59].

When ten women (35 to 65 years old) were treated with 3.2% HCCs and plasma exeresis for neck skin laxity, they experienced 2.4/10 (VAS) mean pain at each session nd crusts that formed resolved within 3 to 7 days. Cutaneous erythema lasted up to 30 days. Mild edema, bruises and ecchymoses were observed [145].

In a study on 50 subjects using a 3.2% HCC body kit, 86% of subjects reported mild or no pain post-treatment and 14% moderate-to-severe pain, but none reported severe or worst pain possible. For 72%, symptoms subsided within 24 h, for 94% within 48 h from the treatment, while just 6% experienced pain ≥72 h post-treatment [35]. In another similar study on 50 women, only light bruising that subsided was reported, with no other adverse events after 4 months of the beginning of the treatment [96]. Furthermore, in another study evaluating 3.2% HCC body formulation for treatment of hand laxity, only bruising was reported. This bruising subsided within 5 to 7 days. No other adverse events were reported 4 months after beginning of the treatment [147].

In a study evaluating 3.2% HCC body formulation for treatment of inner arm and abdomen laxity in 22 women, treatment was evaluated with excellent tolerance by 45% of subjects and with good tolerance by 55%. Minor bruises appeared in some women but subsided within maximum of two weeks [148]. Furthermore, when 3.2% HCC body formulation was administered in one arm of 9 ex-obese women (35–50 years old) experiencing skin laxity, superficial ecchymoses appeared in 3 cases [149].

When HCCs were evaluated in treatment of psoriasis plaques on three subjects, there were no serious adverse events, apart from a single bruising that healed without scarring within 48 h [151]. The 42-year-old woman with atopic dermatitis symptoms persistent on the face that was treated with HCC experienced just light bruising that subsided in 2 days [155]. When a 54-year-old female was treated for a burn scar contracture with jet-injected HCCs, she reported minimal injection pain, while the 36-year-old male treated for scar also with jet-injected HCCs in three separate sessions reported minimal discomfort during treatment [153].

## 7. Limitations of the Available Evidence

The conducted clinical trials are often single-center, with a small number of subjects. This small number of subjects leads to uneven baseline characteristics even after randomization is employed (i.e., block randomization for spilt-face studies). Very often, improvement could be seen but statistical significance could not be established. Moreover, sample size calculation is often missing or calculated based on the occurrence of adverse events; hence, it is possible that with larger sample sizes, significant differences could be observed. Overall, there is a lack of men included in the studies. Due to the nature of the procedure and the specific injection method, blinding of the subjects cannot be guaranteed, only the blinding of the clinicians making the final assessments [1,51,52,66,99]. Many studies lack a control group; additionally, various instrumental methods are frequently used to assess outcomes, making it difficult to directly compare results across studies [4,50,51,66,96].

The exclusion criteria differed significantly among studies, and some did not consider BMI. Great variations in body mass may contribute to skin laxity and other skin changes [129]. Improvement of skin was most often evaluated by two dermatologists with high inter-rater reliability, but in one study evaluating the effect of HCC on skin laxity of the hands, marked differences were observed between the grading provided by a plastic surgeon and a dermatologist, indicating that training and experience may influence such variables [1,21,146]. One study found that plastic surgeons saw the most improvement, followed by dermatologists and then subjects, highlighting differing expectations between professionals and participants [146]. Moreover, in another study, the clinician gave higher grading to the improvement of inner arm laxity compared to the subject [6].

Another source of bias is that several studies are industry-funded or have authors with conflicts of interest [73].

Follow-up of the included studies was up to 6 months, and there was loss of subjects due to follow-up or subjects not completing the procedure in a number of them, which may affect the interpretation of the outcomes [1,2,27,59,132,148].

Some studies were based on subject-reported data only. In such studies, it is possible that subjects provide socially desirable answers [35]. However, in aesthetic medicine, subject satisfaction is likely of greater importance than objective measurement and should be the pillar and focus of research outcomes, as when seeking these procedures, subjects seek improvement in quality of life and social well-being. Mental and emotional health issues such as depression and anxiety due to certain physical traits, burden to hide them and desire for increased confidence are known drivers for subjects seeking these procedures, so studies based only on subject-reported data may carry more relevance in aesthetic medicine than in other fields of medicine [35,157,158,159].

## 8. Conclusions

In vitro studies indicate that HCCs enhance the vitality of fibroblasts, keratinocytes and adipocytes and stimulate production of collagen and elastin. Research using co-cultures of human keratinocytes and dermal fibroblasts show that HCCs accelerate wound closure. Clinical studies show reduction of wrinkle severity, improvement in skin roughness profile and reduction of skin laxity. Improvement in superficial skin hydration was the most pronounced. These effects last up to 6 months after the procedure. The formulation intended for restoration of fat compartments demonstrated reduction of cheek volume loss and improvement in skin thickness. The effects on acne scarring, TEWL and skin elasticity need further research. Subjects reported moderate-to-high satisfaction levels and indicated a willingness to recommend the treatment. According to published data, adverse events occurred in not more than 0.04% of exposed subjects for both products, and were related to injection site conditions, skin conditions and subcutaneous tissue disorders depending on the formulation. Furthermore, HCCs exhibited the lowest hazard ratio for complications among the investigated non-surgical aesthetic treatments, as well as the lowest severe complication rate. In terms of safety, the studies included in this review reported mostly mild pain, edema, bruises and ecchymoses. Regarding safety, most studies reviewed indicated only mild pain, swelling, bruising and ecchymosis. Future research involving multiple centers and patients selected with consistent criteria should provide long-term follow-up results. Additionally, individuals considering this procedure would benefit from data comparing HCCs to other comparable products on the market.

## Figures and Tables

**Table 1:** Overview of in vitro studies with HCCs.

References	Cells	Tested Formulation	Key Findings
[23,33,91]	Keratinocytes and fibroblasts	3.2% HCCs	Increased elastin, type I collagen protein synthesis was higher in fibroblasts than in keratinocytes, increase in HBD-2, reduction in TNF-α and IL-8 protein expression, accelerated scratch closure
[92]	Fibroblasts	3.2% HCCs	Increase in total collagen production
[114]	Mesenchymal stromal cells	3.2% HCCs	Delayed senescence, promoted adipogenic and chondrogenic differentiation
[38]	Human adipose-derived stem cells	3.2% HCCs	Increase in proliferation, induced adipogenic differentiation, induction of strong secretion of adiponectin and leptin
[102]	Human adipose stromal cell	4.5% HCCs	Sustained viability of the cells, faster cell growth compared to H-HA, progressive increase in intracellular lipid droplets

HBD-2—human beta-defensin 2; HCCs—hybrid cooperative complexes of low- and high-molecular-weight hyaluronic acid; H-HA—high-molecular-weight hyaluronic acid; IL-8—interleukin-8; TNF-α—tumor necrosis factor-alpha.

**Table 2:** Characteristics of clinical studies for facial rejuvenation with HCCs.

Reference	Type of Study	Intervention	Control/Comparator	Number of Enrolled Subjects	Number of Subjects Included in Final Analysis	Evaluation or Follow-Up
[1]	Single-blind randomized controlled pilot study	autologous fat transplantation with two injections of 3.2% HCCs two and six weeks after the fat transplantation	only autologous fat transplantation to the cheeks	10 women	9 women	1 month and 6 months postprocedure
[2]	Three case studies	formulation combining hyper-dilute calcium hydroxylapatite, polydeoxyribonucleotide and 3.2% HCCs	N/A	3 women	3 women	6 months postprocedure
[6]	Case report	Three sessions of 3.2% HCCs 30 days apart followed by 100 units of botulinum toxin 30 days after the last HCC application	N/A	1 woman	1 woman	N/A
[6]	Case report	3.2% HCCs at baseline, after 30, 90, 150 and 270 days with cross-linked HA after 60 and 210 days	N/A	1 woman	1 woman	N/A
[27]	Monocentric, open-label, not-controlled, exploratory study	Two applications of 3.2% HCCs 4 weeks apart	N/A	64 women	60 women (of which 4 concluded at 12 weeks)	4, 8, 12 and 16 weeks
[37]	Prospective non-consecutive case series	3.2% HCCs on the left side of the face	Cross-linked HA filler on the right side of the face	15 women	15 women	Before the procedure and every 6–7 days until 3 weeks
[83]	A retrospective evaluation	Two applications of 3.2% HCCs 4 weeks apart	N/A	15 women	15 women	4 and 8 weeks after baseline
[84]	Monocentric observational study	Two applications of 3.2% HCCs 1 month apart	N/A	11 women	11 women	Before the procedure 1 and 2 months after initial treatment
[93]	Open, single-centered, controlled clinical trial	3.2% HCCs at baseline, after 4 weeks, and every 2 months for 7 applications in total	N/A	30 women	23 women	12 months from the first application (4 weeks after the last application)
[99]	Single-center study	Two applications of 4.5% HCCs in the lateral cheek fat compartment 1 month apart	N/A	50 Caucasian women	50 Caucasian women	Before the procedure, after 1 month, and 3 months after the end of treatment
[100]	Case series	Two applications of 4.5% HCCs in the lateral cheek fat compartment 30 days apart	N/A	22 subjects	22 subjects	Immediately after treatment, 3 and after 6 months after first treatment
[125]	Case report	Two applications of 3.2% HCCs 30 days apart	N/A	3 women	3 women	N/A
[128]	Monocentric observational study	Three applications of 3.2% HCCs 4 weeks apart	N/A	10 women	10 women	Before the procedure, after 4, 8 and 12 weeks
[129]	Open-labeled, exploratory, single-center study	Two applications of 3.2% HCCs 4 weeks apart	N/A	30 Asian women	28 women at week 4, 26 women at week 12	Before the procedure, after 4 and 12 weeks
[132]	Single-center clinical trial	Two applications of 3.2% HCCs 4 weeks apart	N/A	10 Chinesewomen	10 women at weeks 4 and 8, 3 women at week 12, none at week 16	Before the procedure, after 4, 8, 12 and 16 weeks

HA—hyaluronic acid; HCCs—hybrid cooperative complexes of low-and high-molecular-weight hyaluronic acid; N/A—not applicable.

## Data Availability

No new data were created or analyzed in this study. Data sharing is not applicable to this article.

## Abbreviations

The following abbreviations are used in this manuscript:

3D	Three-Dimensional
AQP3	Aquaporin 3
BAP	Bio-Aesthetic Points
BTH	Bovine Testicular Hyaluronidase
CUPRAC	Cupric Reducing Antioxidant Capacity
FRAP	Ferric Reducing Antioxidant Power
FVLS	Facial Volume Loss Scale
GAIS	Global Aesthetic Improvement Scale
HA	Hyaluronic Acid
HBD-2	Human Beta-Defensin 2
HCC	Hybrid Cooperative Complex (of Low- and High-Molecular-Weight Hyaluronic Acid)
H-HA	High-Molecular Weight Hyaluronic Acid
IL	Interleukin
kDa	kiloDalton
L-HA	Low-Molecular-Weight Hyaluronic Acid
MMP	Matrix Metalloproteinase
MW	Molecular Weight
ORAC	Oxygen Radical Absorbance Capacity
RHAMM	Receptors For Hyaluronan-Mediated Motility
ROS	Reactive Oxygen Species
TEWL	Transepidermal Water Loss
TGF-β1	Transforming Growth Factor Beta 1
TLR	Toll-Like Receptors
TNF-α	Tumor Necrosis Factor-Alpha
TSASRT	Triple-Step Acne Scar Revision Technique
VAS	Visual Analogue Scale
WSRS	Wrinkle Severity Rating Scale
